# Exploring New Strategies for Optimizing the Production of Poly(3-hydroxybutyrate-*co*-3-hydroxyvalerate) from Methane and VFAs in Synthetic Cocultures and Mixed Methanotrophic Consortia

**DOI:** 10.1021/acssuschemeng.3c08570

**Published:** 2024-03-04

**Authors:** Claudia Amabile, Teresa Abate, Enrique Marcos, Simeone Chianese, Dino Musmarra, Raul Muñoz

**Affiliations:** † Department of Engineering, University of Campania “Luigi Vanvitelli”, Via Roma 29, Aversa 81031, Italy; ‡ Institute of Sustainable Processes, University of Valladolid, Dr. Mergelina, s/n, Valladolid 47011, Spain

**Keywords:** biopolymers, poly(3-hydroxybutyrate-*co*-3-hydroxyvalerate), sustainable process, mixed methanotrophic consortium, synthetic cocultures, volatile fatty acid mixture

## Abstract

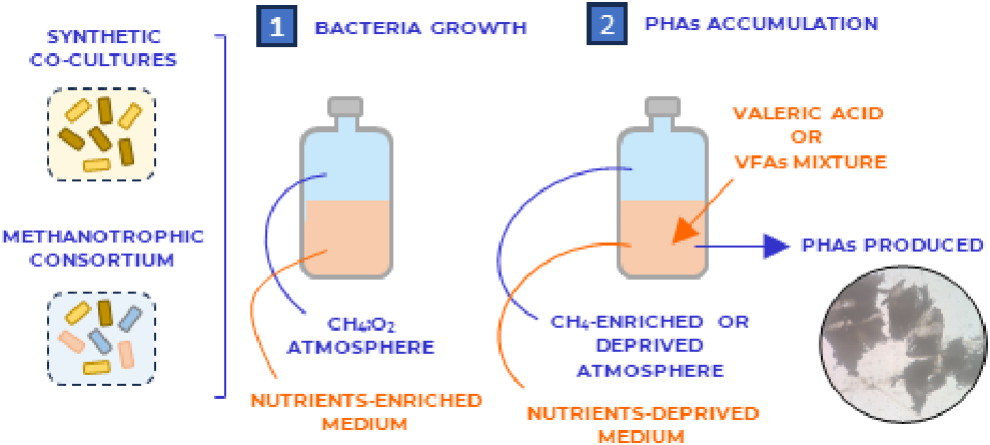

In this work, the potential of a synthetic coculture and a mixed methanotrophic consortium to synthesize poly(3-hydroxybutyrate-*co*-3-hydroxyvalerate) (PHBV) from renewable and waste-based feedstocks was assessed batchwise. *Methylocystis parvus*cocultivated with *Rhodococcus opacus* and a *Methylocystis*-enriched culture previously grown on methane were subjected to nutrient starvation in a medium enriched with valeric acid (30% w w^–1^ of *C*_tot_) or with a VFAs mixture containing acetic, propionic, butyric, and valeric acids (15% w w^–1^ of *C*_tot_) under a CH_4_:O_2_ or air atmosphere. For all test series, pH was adjusted to 7 after adding the cosubstrates, and a negligible substrate consumption or polymer production was considered the end point of the trial. Results showed that valeric acid promoted PHBV accumulation in both cultures regardless of the atmosphere. Interestingly, the mixture of VFAs supported PHBV accumulation only in the presence of methane. The highest PHBV contents for the coculture and the mixed consortium, equal to 73.7 ± 2.5% w w^–1^ and 49.6 ± 13% w w^–1^, respectively, were obtained with methane and the VFAs mixture. This study demonstrates the suitability of cocultures and biobased cosubstrates for the sustainable production of the biodegradable polymer PHBV.

## Abstract 2

The development of an effective strategy for producing biodegradable polymers using renewable resources could enable the shift toward sustainable materials.

## Introduction

Biobased and biodegradable polyhydroxyalkanoates (PHAs) have attracted a recent interest in the scientific community based on their properties, which are very similar to those of conventional plastics such as polypropylene or polystyrene.^1,2^ To date, more than 55 species of Gram-positive and Gram-negative bacteria have been identified as PHAs producers under the absence or deficiency of nutrients such as nitrogen, phosphorus, or potassium; other microorganisms such as cyanobacteria and haloarchaea have also been successfully tested. The suitability of the microorganism considered depends on the ability of the strain to metabolize a primary carbon source under nutrient deficiency, some of them being particularly suitable, thanks to their versatility and adaptability to several cultivation conditions.^3^ Under these metabolic stress conditions, bacterial strains which are PHAs producers are able to use a carbonaceous substrate to produce intracellular PHAs for further use as carbon and energy reservoir.^4,5^ The polyesters synthesized are accumulated in the form of insoluble granules in the cell cytoplasm until mobilization.^6,7^ Since the first identification of PHAs, many monomers forming these biodegradable polymers have been discovered. For instance, Wallen and Rohwedder in 1974 obtained from activated sewage sludge polymers 3-hydroxyvalerate (3-HV), 3-hydroxyhexanoate (3-HHx), and 3-hydroxyheptanoate (3-HHp) monomers.^8^ To date, 150 different monomers and 120 types of PHAs have been identified.^9^ The differences among all the existing PHAs lie in the type of monomer units, which can range from 100 to 30000, in the content of −CH_2_– and in the alkylic group (it can be either linear or branched, saturated or unsaturated, etc.).^10^ Depending on the length of their monomers, PHAs can also be classified as (i) short-chain-length PHAs (SCL-PHAs), when the alkylic group has less than 5 carbon atoms; (ii) medium-chain-length PHAs (MCL-PHAs), when the number of carbon atoms ranges from 5 to 14; and (iii) long-chain-length PHAs (LCL-PHAs), with more than 14 atoms in the alkylic group.^11,12^ Overall, the molecular structure of the PHAs determines their properties and potential applications.

Biodegradable PHAs are promising candidates to replace conventional and fossil-based plastics, which are causing severe environmental problems nowadays. Indeed, about 350 million tons of plastic waste are generated every year worldwide, and its production is foreseen to triple by 2060.^13^ Since plastics could take more than 500 years to decompose, depending on their structure and composition, a valid solution providing the same range of applicability for large-scale production should be found promptly.^14,15^ In this context, PHAs are nowadays used in packaging, agriculture (encapsulation of seeds, fertilizers for slow-release, biodegradable plastic films for crop protection, and containers for hothouse facilities), electronics, nanotechnology (nanoparticles, nanocapsules, microparticles, microcapsules, and micro/nanospheres for drug delivery), transplantology, tissue engineering, and pharmacology.^16,17^ However, the technology readiness level (TRL) of PHAs production is still low (<5), thus implying that a deep analysis of the production mechanisms and the optimization of process yields are still required to promote the large-scale application of these biodegradable polyesters.^18,19^ Among all PHAs, poly(3-hydroxybutyrate) (PHB) has been the most studied, while poly(3-hydroxybutyrate-*co*-3-hydroxyvalerate) (PHBV), which results from the combination of 3-HB and 3-HV monomers, has been included in the literature among the best performing polymers belonging to the category of PHAs.^20,21^ Indeed, the properties of PHBV make it very similar to commercial plastics, such as polypropylene, thus allowing its application in a wide range of sectors. Compared to PHB, PHBV is more resistant and has a higher elongation to break and a lower melting temperature. Moreover, the lower crystallinity of PHBV makes its biodegradation process faster than that of PHB (Table 1).^22,23^

**Table 1:** Comparison of the Properties of PHB, PHBV, and PP^5,24−26^

	PHB	PHBV	PP
*M*_n_	1.48 × 10^6^	6.7 × 10^5^ to 1.33 × 10^6^	-
*T*_m_ [°C]	177–178	145–168	176
*T*_g_ [°C]	3–4	–0.8 to −2	–10
*E* [GPa]	3–3.5	1–1.2	0.004
σ_t_ [MPa]	40–43.2	22–45.6	-
ε [%]	3.5–5.2	50–176	550
crystallinity [%]	60	40–60	50

However, while PHB can be produced from a variety of substrates, the synthesis of 3-HV requires specific precursors.^27^ Volatile fatty acids (VFAs) have been reported as effective cosubstrates during the production of PHBV, with valeric acid showing the best performance in terms of 3-HV synthesis.^28,29^

To date, only a few experimental studies aimed at optimizing the production of PHBV, and the scale-up of this process is still a challenge since it requires enhanced PHBV productivity and a reduction in production costs.^27^ Indeed, the carbon sources conventionally used for PHBV production can represent up to 50% of the total process cost.^30,31^ In this regard, the use of methane as a substitute for expensive oils and sugars and the supply of volatile fatty acids derived from the fermentation of organic waste instead of pure valerate could eventually reduce PHBV production costs.^28,32,33^ The conversion of these carbonaceous substrates into PHAs can be performed by specific microorganisms known as type II methanotrophs.^34^ Pure strains such as *Methylocystis hirsuta* and *Methylocystis parvus* yield the highest PHAs accumulation, but the cultures have shown severe metabolic instability and only PHB can be produced when using methane as a carbon and energy source.^35−37^ In this context, the use of mixed methanotrophic consortia or synthetic cocultures supplemented with VFAs could overcome metabolic instability and simultaneously improve PHBV productivity from methane.^38,39^ In this study, a novel strategy for producing PHBV in a sustainable and economically viable way was proposed. First, the possibility of using synthetic cocultures to lower the sterility requirements, create a more favorable cultivation environment, and enhance the production yields was investigated. Indeed, despite mixed consortia work under aseptic conditions, the PHA productivity is generally lower than that of the pure strains. Therefore, *M. parvus* was selected as the main PHA producer, and *Rhodococcus opacus* was added as partner microbe for cocultivation since the combination of these two strains resulted in a synergistic effect among the species.^40^ Valeric acid was fed as a cosubstrate for triggering 3-HV formation. Then, the synthesis of 3-HV monomers, both in the presence and absence of methane, was studied by replacing pure valeric acid with a mixture of four volatile fatty acids (acetic, butyric, valeric, and propionic acids) that simulated the mixture extracted from a real effluent of the hydrolytic fermentation of food waste at 35 °C. In this context, it should be stressed that using residual effluents could notably lower the costs for the cosubstrates. Finally, the same experimental setup was replicated by replacing the synthetic consortium with a naturally developed mixed methanotrophic culture to compare the yields obtained with those of the two cultures and validate the PHBV enhancement strategy proposed.

## Materials and Methods

### Chemicals

Culture media were prepared by using chemicals acquired from PANREAC AppliChem (Barcelona, Spain) and Sigma-Aldrich and Labkem (Barcelona, Spain). Valerate (≥99%), propionate (≥99%), butyrate (≥99%), and acetate (99%), which were used for preparing cosubstrate solutions, were purchased from Sigma-Aldrich. Chloroform (≥99.8%), 1-propanol (99.7%), benzoic acid (99.5%), hydrochloric acid (37% w v^–1^), and poly(3-hydroxybutyrate-*co*-3-hydroxyvalerate) (PHBV, with 3-HV molar content of 12%) (99.99%), which were used for polymer extraction and quantification, were purchased from Sigma-Aldrich. O_2_ (99.5%) and CH_4_ (99.5%) were obtained from Abelló Linde S.A. (Spain) and Carburos Metallicos (Spain), respectively.

### Strains and Culture Medium

*Methylocystis parvus* OBBP (Biopolis S.L., Valencia, Spain) and a *Methylocystis*-enriched consortium obtained according to a previous study were used as methanotrophic PHA producers.^32^ The mixed consortium used was obtained from *Sphagnum* and dominated by the genus *Methylocystis* (>50%).^32^ Both strains were grown in a mineral salt medium (NMS) containing macronutrients (g L^–1^): 0.2 CaCl_2_·2H_2_O, 1.0 KNO_3_, and 1 mL of a trace element solution composed of (mg L^–1^) 0.38 Fe-EDTA, 0.4 Na_2_MoO_4_·2H_2_O, 0.3 Na_2_EDTA·2H_2_O, 1.0 CuSO_4_·5H_2_O, 0.5 FeSO_4_·7H_2_O, 0.4 ZnSO_4_·7H_2_O, 0.015 H_3_BO_3_, 0.01 and 0.03 CoCl_2_, 0.02 MnCl_2_·4H_2_O, and NiCl_2_·6H_2_O. The addition of 10 mL of a buffer solution containing 72 g L^–1^ Na_2_HPO_4_·12H_2_O and 26 g L^–1^ KH_2_PO_4_ was required to set the initial pH at 6.8.

The culture medium described above, deprived of KNO_3_, was used for the PHBV production stage. During this phase, pure valerate and a mixture of VFAs (36.5% acetate, 31.7% propionate, 21.9% butyrate, and 9.9% valerate) were supplied as cosubstrates for producing PHBV. It is worth noting that the composition of the VFA mixture aimed to replicate a mixture that can be recovered from the digestate produced during the anaerobic digestion (AD) of food waste, in which the specific operating conditions of temperature (*T*: 35 °C) and pH (5–7) allowed to incorporate up to 10% of valeric acid, which is a pivotal and well-established precursor for 3-HV inclusion.^41^ Both cosubstrates were supplied to represent 15% and 30% of the total carbon (*C*_tot_) present in the bottles, respectively. The fraction of carbon supplied through the cosubstrate was determined based on previous experimental studies.^42^ More specifically, it was demonstrated that a concentration of cosubstrate around 15% is sufficient for inducing 3-HV production, while 30% can be set as the upper tolerance limit for the biomass involved, at which the copolymer formation does not further increase.

*Rhodococcus opacus* DSM 43205 (Leibniz Institute, Germany), used as a microbial partner during *M. parvus* cultivation, was grown in M9 mineral salt medium consisting of (g L^–1^) 7.52 Na_2_HPO_4_·2H_2_O, 3 KH_2_PO_4_, 0.5 NaCl, 0.5 NH_4_Cl, 4 C_6_H_12_O_6_, 0.246 MgSO_4_·7H_2_O, 0.044 CaCl_2_·2H_2_O, and vitamins (1 mL of biotin and 1 mL of thiamine solutions). A trace element solution (10 mL L^–1^) containing (g L^–1^) 5 EDTA, 0.83 FeCl_3_·6H_2_O, 0.084 ZnCl_2_, 0.013 CuCl_2_·2H_2_O, 0.01 CoCl_2_·2H_2_O, 0.01 H_3_BO_3_, and 0.0016 MnCl_2_·4H_2_O was also added. *Rhodococcus opacus* DSM 43205 was selected based on its ability to degrade a wide range of organic compounds, including some secondary byproducts of the metabolism of *M. parvus* that could potentially inhibit its growth.

## Experimental Procedures

### Inocula Preparation

*M. parvus* and the mixed methanotrophic consortium (MMC) were first cultivated into sterile 125 mL serum bottles containing 50 mL of MSM (10% v v^–1^). The bottles were capped with butylrubber stoppers and crimp-sealed before incubation at 200 rpm and 25 °C for 6 days. Methane was provided via injection of a CH_4_:O_2_ mixture (33.3:66.6% v v^–1^) into the headspace. First, pure oxygen was continuously supplied into the headspace of the serum bottle for 4–5 min; then, 25 mL of the atmosphere was replaced with pure methane using a 50 mL gastight syringe (Hamilton 1050 TLL, USA). The atmosphere was renewed every 48 h, until a high cell density was achieved. Sterile conditions were required only for pure cultures. An aliquot of 2 mL of *R. opacus* stock inoculum was resuspended in 20 mL of M9 mineral salt medium under strictly sterile conditions and incubated at 250 rpm and 25 °C for 48 h. Then, the *R. opacus* active culture was scaled up by resuspending 20 mL of the active inoculum in a 2.15 L bottle containing 0.5 L of M9 medium and stirring at 300 rpm and 25 °C for 3 days. Once all cultures were active, sterile 2.15 L serum bottles containing 0.5 L of NMS were inoculated with 10 mL of the active cultures of *M. parvus* and 10 mL of *R. opacus* for the coculture experiment or with 20 mL of the MMC. *R. opacus* broth was centrifuged at 4 200 rpm for 10 min before the inoculation to remove residual ammonium or glucose. After the inoculation, which took place under a CH_4_:O_2_ atmosphere with a ratio of 33.3:66.6% v v^–1^), the bottles were placed on multipoint stirrers (Variomag, Thermo Fisher Scientific, USA) and agitated at 300 rpm and 25 °C until methane was almost completely depleted.

### PHBV Synthesis in *M. parvus* Cocultures and Mixed-Methanotrophic Consortium

**Figure 1:**
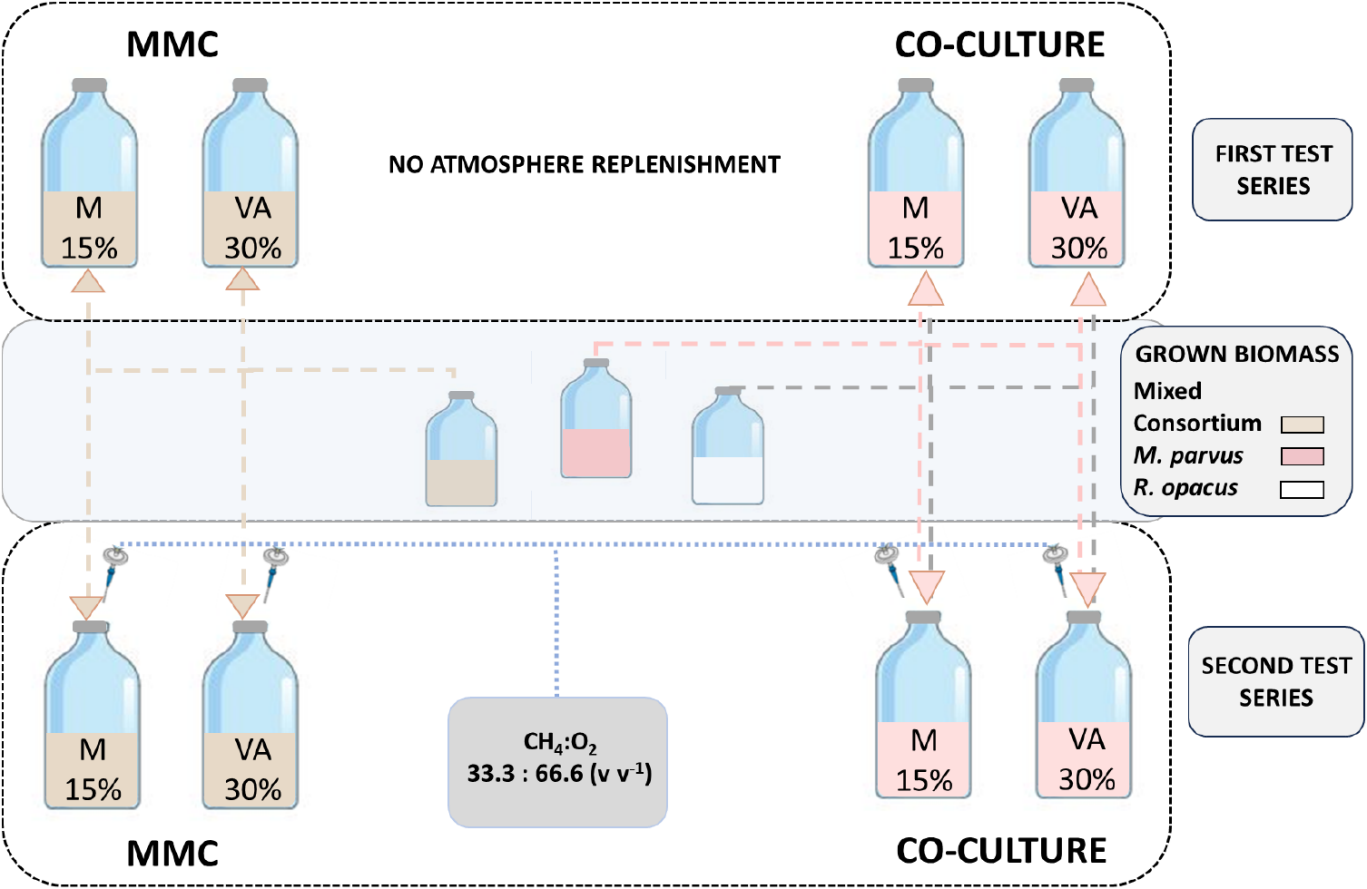
Overview of test series.

When methane was almost completely depleted during the growth phase, all culture broths were centrifuged at 4200 rpm for 10 min to harvest the pellet of *M. parvus* + *R. opacus* and the mixed consortium. PHBV accumulation tests were then performed by resuspending the solids into 2.15 L serum bottles containing 0.5 L of nitrogen-free mineral salt medium. Since 3-HV precursors are needed to induce PHBV synthesis, the medium was supplemented with a valeric acid concentration representing 30% w w^–1^ of the total carbon in the bottles or a synthetic mixture of VFAs (M) representing 15% w w^–1^ of the total carbon. pH was manually controlled and adjusted to 7 during all test series by withdrawing 5 mL of culture medium, which served to estimate the amount of base/acid to be added to the bottles. For both cultures, tests were performed in duplicate under two conditions: (i) absence of methane in an air headspace and (ii) a CH_4_:O_2_ = 33.3:66.6% v v^–1^ headspace. The bottles were incubated at 350 rpm and 25 °C on a multipoint stirrer. The concentration of TSS, the composition of the headspace, the content of PHAs, and their composition were monitored every 48 h. An overview of the experimental plan is shown in Figure 1.

### Analytical Methods

The composition in the headspace of the bottles (methane, oxygen, and carbon dioxide) was measured every 48 h using a Bruker 430 GC-TCD (Bruker Corporation, Palo alto, USA) equipped with a CP Molsieve 5A and a CP-PoraBOND Q columns. The optical density (OD) at 600 nm was measured using a UV-2550 spectrophotometer (Shimadzu, Japan), while total suspended solid concentration was estimated according to the 2540 standard method.^43^ Samples for VFA quantification in the liquid phase were prepared by filtration of 1 mL of culture broth and further acidification with 30 μL of H_2_SO_4_. Samples were stored at 4 °C until analysis in an Agilent 7820A GC-FID instrument (Agilent Technologies, Santa Clara, USA). Temperatures of the oven, injector, and detector were kept at 130, 375, and 350 °C, respectively. For PHB/PHBV extraction, samples of 1.5 mL of culture broth were centrifuged at 10000 rpm for 10 min to harvest the pellet (*n* = 3), which was stored at −20 °C until use.^37^ PHB/PHBV analysis and quantification were performed via gas chromatography–mass spectrometry using a 7820A GC coupled with a 5977E MSD instrument (Agilent Technologies, Santa Clara, USA) and equipped with a DB-wax column. Benzoic acid in propanol (40 g L^–1^) and PHBV (12%mol HV) were used as internal and external standards, respectively.

## Results and Discussion

### Growth and PHBV Accumulation in *M. parvus* Cocultures and MMC Under Valeric Acid Supplementation and Methane Deprivation

**Figure 2:**
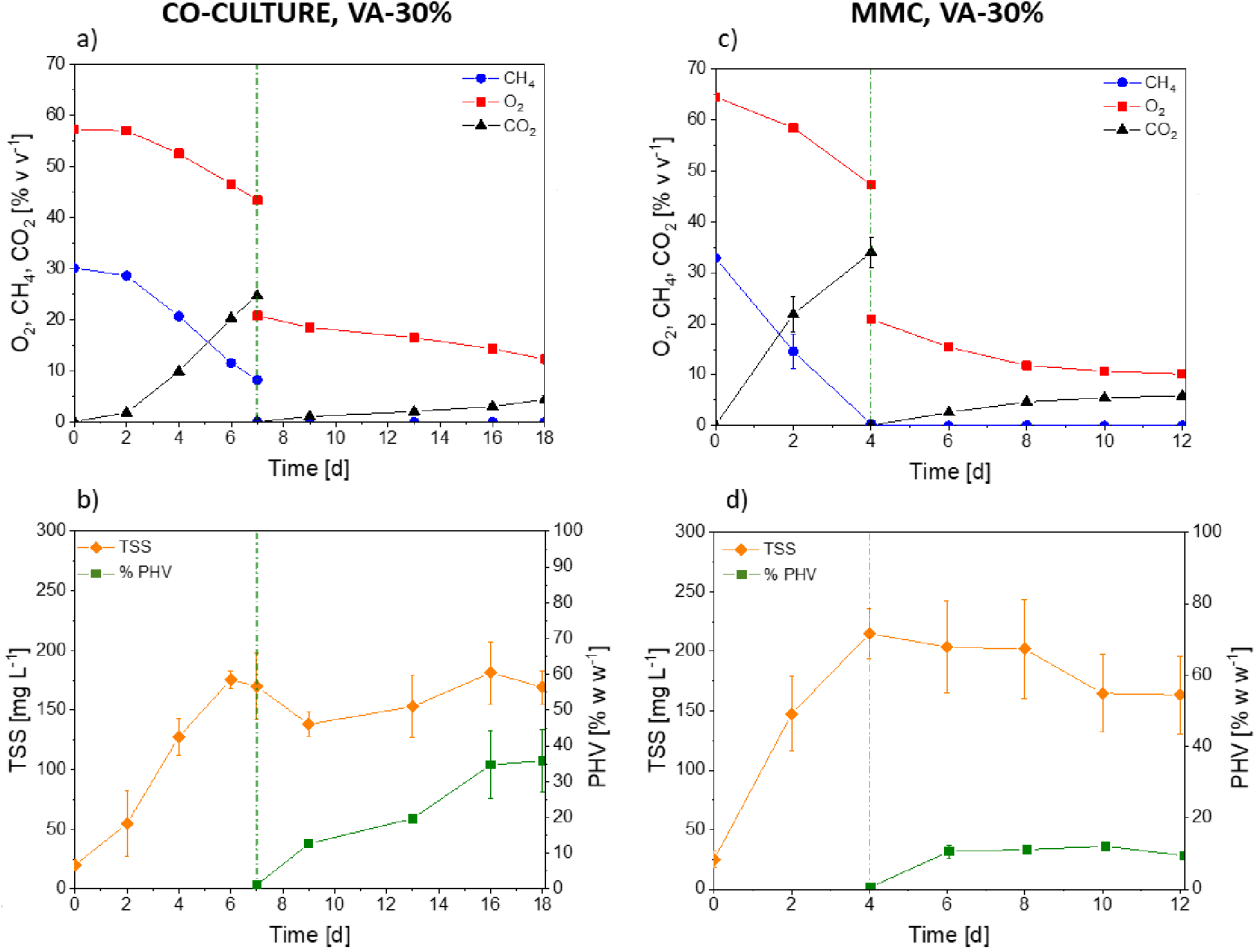
Time course of oxygen, methane, carbon dioxide, TSS, and PHA concentrations in the tests conducted with *M. parvus* + *R. opacus* cocultures (a, b) and the mixed methanotrophic consortium (c, d) in a medium supplemented with valeric acid (30% w w^–1^ of *C*_tot_). No methane was supplied during PHBV accumulation (a, c).

The ability of synthetic cocultures of *M. parvus* and *R. opacus* and the methanotrophic mixed consortium to accumulate PHBV under a methane-deprived atmosphere with precursor (valeric acid) supplemented at 30% w w^–1^ of the total carbon was evaluated. The cocultures (initial OD_600_ = 0.1) required 7 days to reach a concentration of 170 ± 28 mgTSS L^–1^ and use 72.6 ± 0.7% v v^–1^ of CH_4_ and 24.2 ± 0.4% v v^–1^ of O_2_ initially supplied (Figure 2a,b). The total CO_2_ production during the growth phase accounted for 486 ± 8 g m^–3^. The cultivation of the mixed methanotrophic consortium (initial OD_600_ = 0.25) led to higher methane and oxygen consumptions of 99 ± 7% v v^–1^ and 26.7 ± 0.6% v v^–1^, respectively, while 667.6 ± 7 g m^–3^ CO_2_ wasgenerated (Figure 2c). Accordingly, a higher final cell concentration of 215 ± 21 mgTSS L^–1^ was reached (Figure 2d). Moreover, the MMC only required 4 days to deplete all the carbon present in the headspace, thus suggesting that the cultivation conditions during the growth phase were more suitable for the MMC than for the cocultures.

**Figure 3:**
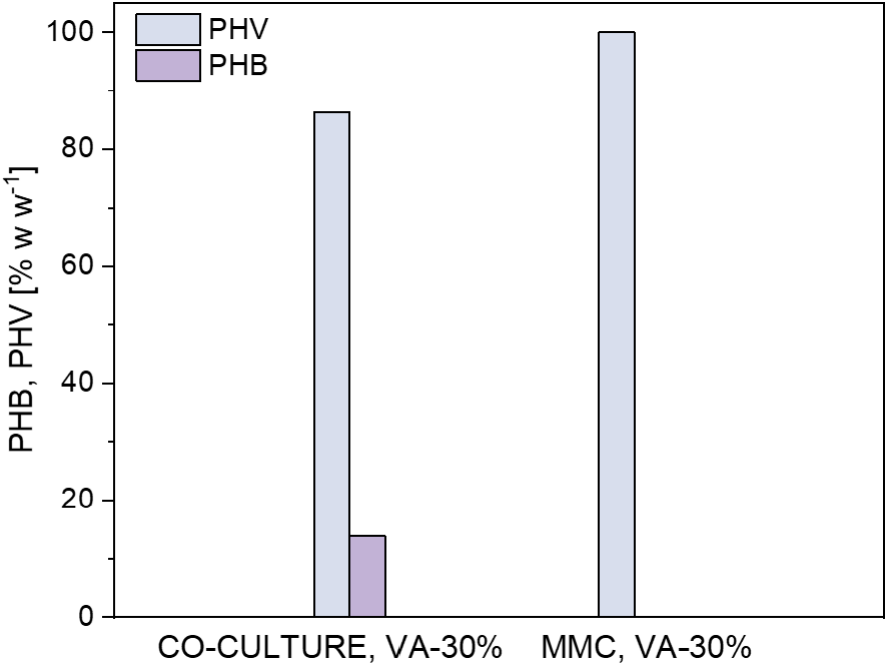
Average PHAs composition obtained with valeric acid supplemented at 30% w w^–1^ of the total carbon during the cultivation of synthetic cocultures and the mixed methanotrophic consortium.

When methane depletion was stopped, both cultures were resuspended into a nitrogen-free MSM enriched with valeric acid in the absence of methane. The synthetic cocultures entailed the consumption of 40.8 ± 4% v v^–1^ of the initial O_2_ and a CO_2_ production of 87 ± 16 g m^–3^ by day 18 (Figure 2a). This metabolic activity was linked to a maximum PHBV production of 35.9 ± 8.6% w w^–1^ by day 18, with the PHV fraction accounting for 80% of the total polymer synthesized (Figure 2b). The highest PHV content (86.2% of the PHBV) was obtained by day 16, thus suggesting that from this moment onward the metabolic pathway switched to the synthesis of PHB instead of PHV (Figure 3). A similar polymer composition was previously reported by Lopez et al. (2018), who cultivated *Methylocystis hirsuta* with valeric acid as the sole carbon and energy source and obtained PHBV with a 3-HB:3-HV molar ratio equals to 17:83. However, the production yields in that study accounted for only ≈9% w w^–1^, which was 4-fold lower than the productivity achieved with the cocultures in this work. Although PHA production is known to be strain dependent, the use of a microbial partner during the cultivation of *M. parvus* could mediate synergisms among the species involved. Indeed, Myung et al. (2016) cultivated *M. parvus* under a methane-deprived atmosphere with valeric acid as a cosubstrate at 100 ppm without any PHA accumulation in the cells. These considerations reinforced the hypothesis that cocultures could create a more favorable environment for PHAs accumulation in type II methanotrophs.

MMC cultures were resuspended by day 4 in a nitrogen-deprived medium supplemented with valeric acid in the absence of methane. Under nitrogen limiting conditions, consumption of 52.6 ± 2.6% v v^–1^ of the total O_2_ fed and a CO_2_ production of 117.4 ± 1.9 g m^–3^ were observed by day 14 (Figure 2c). A maximum PHA content of 12 ± 1% w w^–1^ with a PHB:PHV ratio of 0:100 was observed by day 10 (Figure 3). Interestingly, MMC cultures only synthesized PHV; while the synthetic coculture was able to generate both PHB and PHV in the absence of methane. *Methylosinus*-dominated cultures, which were studied by other authors, accumulated a similar PHA content (8.5% w w^–1^) with a 3-HV fraction of 56% w w^–1^ under similar conditions used in this work.^44^ This finding suggests that the composition of PHAs, in addition to being dependent on the cultivation conditions, is also determined by the type of microorganisms. In the particular case of MMC, these differences could be related not only to the dominant genus but also to the strains in cocultivation and their specific enzymatic activity. Finally, during this test series, the PHA yields of MMC were 3 times lower than those recorded in the cocultures, which suggested the suitability of cocultures for reducing the needs of sterilization and enhancing process yields during cultivation with valerate as the sole carbon and energy source.

### Growth and PHBV Accumulation in *M. parvus* Cocultures and MMC Under a VFA Mixture Supply and Methane Deprivation

**Figure 4:**
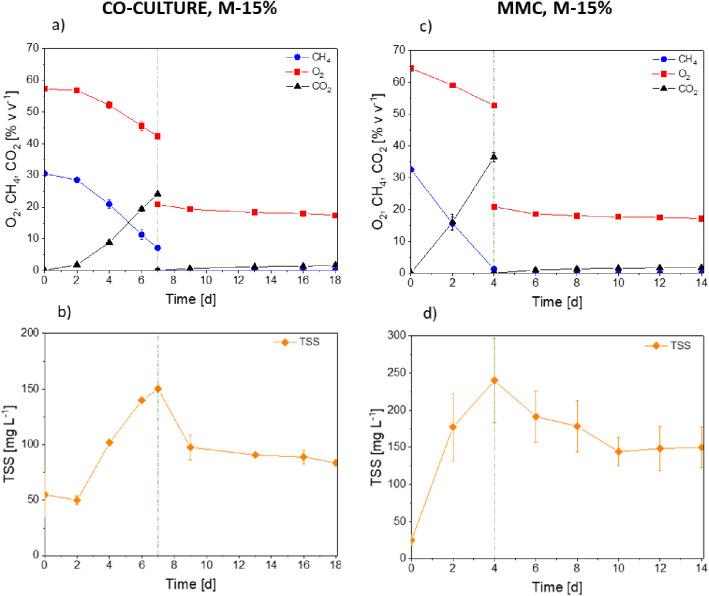
Time course of oxygen, methane, carbon dioxide, and TSS concentrations in *M. parvus* + *R. opacus* cocultures (a,b) and the mixed methanotrophic consortium (c,d). No methane was supplied during starvation (a, c).

The potential of mixtures of valeric, acetic, propionic, and butyric acids simulating real effluents extracted from the mesophilic fermentation of food waste to foster PHBV synthesis in the absence of methane was assessed. The cocultures (initial OD_600_ = 0.1) were first grown for 7 days with an associated use of 76.7 ± 2.6% v v^–1^ of CH_4_ and 26 ± 1.6% v v^–1^ of O_2_ initially present in the headspace (Figure 4a). The final TSS concentration and the net CO_2_ production accounted for 150 ± 0 mgTSS L^–1^ (corresponding to an OD_600_ of 1.75) and 473.8 ± 2 g m^–3^, respectively, by day 7 (Figure 4b). Similar to the assays conducted with valeric acid, the cultivation of MMC (initial OD_600_ = 0.19) led to a higher cell concentration up to 270 ± 56 mgTSS L^–1^ with an associated CO_2_ production of 716 ± 26 g m^–3^ (Figure 4d). Compared to the cocultures, the methane used by MMC increased to 96.2 ± 2% v v^–1^ while only 18 ± 0.6% v v^–1^ of the oxygen initially supplied was consumed.

The synthetic cocultures and the MMC were resuspended in a nitrate-free medium containing the VFA mixture at a concentration of 15% w w^–1^ of the total carbon by days 8 and 4, respectively (Figure 4a,c). During the nitrogen starvation period, cultures of *M. parvus* + *R. opacus* supported the elimination of only 16.5 ± 0.6% v v^–1^ of the oxygen initially present in the headspace and the generation of 33.9 ± 0.6 g m^–3^ CO_2_ by day 18 (Figure 4a). In the assays conducted with MMC, similar O_2_ removal of 17.7 ± 1.6% v v^–1^ and carbon dioxide concentration of 34.4 ± 0.4 g m^–3^ were obtained (Figure 4c). The lower oxygen consumption and CO_2_ production observed in this case study could explain the absence of PHA accumulation observed in these assays. Indeed, no polymer accumulation was detected, and the total suspended solid concentration decreased during the nitrogen starvation phase. More specifically, the total suspended solid concentration decreased from 150 ± 0 mgTSS L^–1^ by day 0 to 83 ± 3 mgTSS L^–1^ by day 7 in the tests conducted with cocultures and from 270 ± 56 mgTSS L^–1^ by day 0 to 150 ± 27 mgTSS L^–1^ by day 4 in the assays carried out with the mixed methanotrophic consortium (Figure 4c,d, respectively). These findings could be explained by the unfavorable environmental conditions generated in the absence of a primary carbon source (i.e., methane) in a medium supplemented with the VFA mixture. In this context, the occurrence of other VFAs and the lower concentrations of valeric acid likely governed the inhibition of PHA synthesis into the bacterial biomass compared to the previous test performed only with valeric acid. The consistency of the results obtained in the cocultures and the MMC suggested that supplementing valeric acid above a certain threshold concentration is the only applicable strategy to activate the pathway of PHA synthesis without a primary carbonaceous substrate such as methane. To the authors’ knowledge, no previous studies aimed at PHBV production were conducted using mixtures of volatile fatty acids or cocultivation systems. A study conducted by Lopez et al. (2018) reported that *M. hirsuta* was able to accumulate very low concentrations of PHAs, mainly constituted by PHB, using acetic (2.4% w w^–1^), butyric (1.8% w w^–1^) and propionic (1.1% w w^–1^) acids in the absence of methane. No PHA synthesis was observed during the cultivation of *M. parvus* in propionic acid as the sole carbon and energy source.^36^ Similarly, a mixed consortium was only capable of accumulating a PHA content of 2.8% w w^–1^ (20.5 mol% 3-HV) with propionic acid as the sole carbon source.^29^

### Growth and PHBV Accumulation in *M. parvus* Cocultures and MMC Under a Methane/Oxygen Atmosphere in a Medium Enriched with Valeric Acid

The potential of *M. parvus* + *R. opacus* cocultures and a mixed methanotrophic consortium to accumulate PHBV from methane and valeric acid was evaluated during test series 2. Cultures were grown up to 220 ± 0 mgTSS L^–1^ (coculture, initial OD_600_ = 0.11) and 265 ± 7 mgTSS L^–1^ (MMC, initial OD_600_ = 0.21) prior to resuspension in a nitrate-free medium. During the growth stage, the cocultures supported the use of 80.6 ± 1% v v^–1^ of CH_4_ and 33.5 ± 5% v v^–1^ of O_2_ initially supplied by day 7, while a reduction of 98 ± 0.6% v v^–1^ of CH_4_ and 19.5 ± 3% v v^–1^ of O_2_ was recorded during the first 4 days of cultivation of the MMC. The total net carbon dioxide production in MMC cultures was higher than that of the coculture, accounting for 738 ± 65 g m^–3^ and 460 ± 16 g m^–3^, respectively.

**Figure 5:**
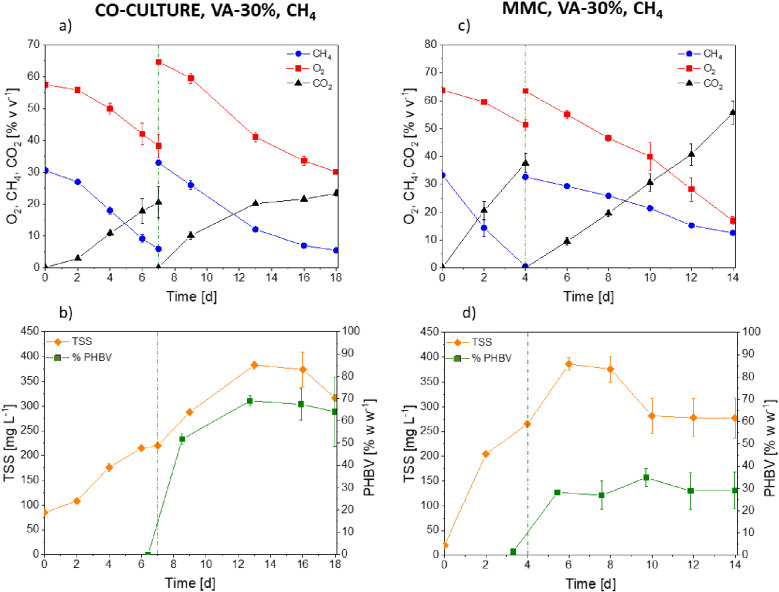
Time course of oxygen, methane, carbon dioxide, TSS, and PHA concentration in *M. parvus* + *R. opacus* cocultures (a, b) and the mixed methanotrophic consortium (c, d) in a medium supplemented with valeric acid (30% w w^–1^ of *C*_tot_).

**Figure 6:**
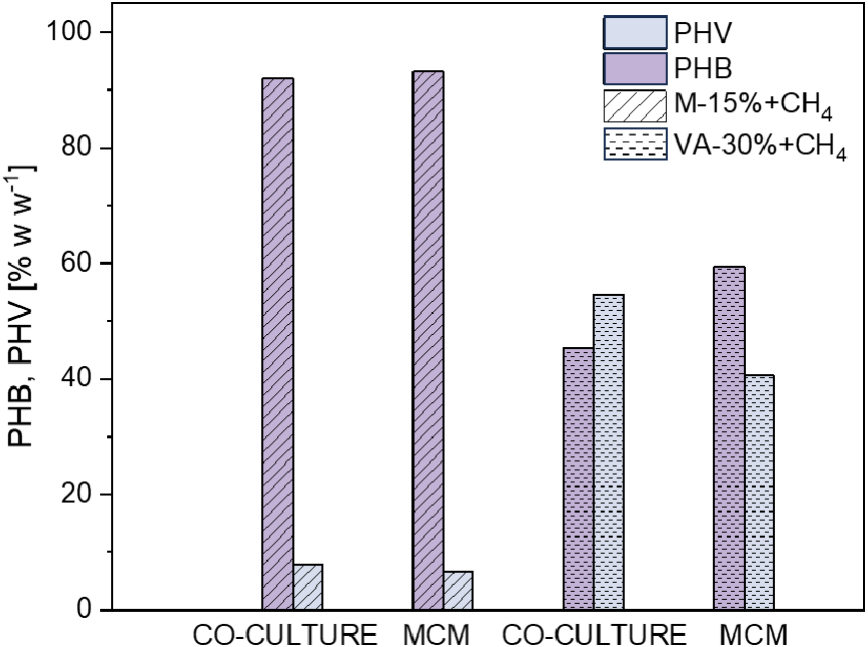
Average composition of PHA obtained in synthetic cocultures and mixed methanotrophic consortium with methane as the primary carbon source in media supplemented with valeric acid and a VFA mixture.

*M. parvus* based cocultures were resuspended by day 7 in a nitrogen-deprived medium under a methane atmosphere with valeric acid. During the nitrogen starvation stage, a similar methane consumption of 80.6 ± 1% v v^–1^ was observed, while the removal of oxygen increased up to 53.4 ± 0.5% v v^–1^ by day 18 (Figure 5a). This behavior confirmed the previous findings of high metabolic-energy requirements during microbial cultivation in the valerate supplemented medium. Indeed, the assimilation of valeric acid has been reported as an energy-intensive process, where a high number of moles of oxygen is required to co-oxidize methane during PHBV generation.^23,28^ In this case, a maximum content of 68 ± 7% w w^–1^ of PHBV was measured by day 16 (Figure 5b), with a PHB:PHV ratio of 45:55 (Figure 6). This is likely the highest PHV content obtained in systems cultivating methanotrophs with valeric acid under a methane atmosphere. For example, a previous study reported that using biogas and valeric acid allowed the production of ≈54% w w^–1^ of PHBV with a 3-HV molar content of 25%.^28^ Likewise, the cultivation of *M. parvus* with methane and valerate (100 ppm) led to ≈54% w w^–1^ of PHBV with a 3-HV molar fraction of 22%.^23^ The results reported in this study confirm the advantages of using cocultivation systems, where the presence of *R. opacus* in cultures of *M. parvus* not only improves the total polymer yields but also increases the PHV fraction stored in the cells. The advantages of synthetic cocultures could also be observed over the mixed methanotrophic consortia. It is worth noting that, since cocultures can yield higher levels of PHAs compared to mixed consortia, have the ability to include 3-HV building blocks, and do not require high sterility conditions, their application could favor the widespread production of PHAs from methane at the industrial level.

In this study, the metabolic stress induced in MMC induced a maximum PHBV content of 35 ± 4% w w^–1^ by day 10 (Figure 5d), with a PHB:PHV ratio of 64:34 (Figure 6). The highest PHV content was detected by day 6 (41% PHV), thus suggesting that in this case the metabolism was mainly devoted to the generation of PHB from day 6 onward. During this stage, the mixed methanotrophic consortium consumed 62 ± 2% v v^–1^ of methane and 73.3 ± 2.5% v v^–1^ of oxygen, which were the lowest and the highest consumptions observed in this work, respectively (Figure 5c). A concomitant carbon dioxide production resulting in a staggering concentration of 1093 ± 83 g of CO_2_ m^–3^ was also observed (Figure 5c), thus suggesting that most of the carbon was converted into CO_2_ instead of PHA. It is worth highlighting that these behaviors are typically observed in mixed methanotrophic consortia, where a high fraction of non-PHA-producer-methane utilizer strains is present in the culture. Similar results were obtained in a previous study, with the achievement of ≈30% w w^–1^ of PHA with approximately 40% mol mol^–1^ of 3-HV by a *Methylocystis*-dominated enrichment in the presence of methane and 400 mg L^–1^ of valeric acid.^23^ Since the results obtained in this work with the MMC are comparable to those reported previously in the literature, it can be stated that the high PHBV yields achieved in cocultivation systems are not related to the external environmental conditions during cocultivation (such as temperature or pH) but depend on the combination of different cellular environments.

### Growth and PHBV Accumulation in *M. parvus* Cocultures and MMC Under a Methane/Oxygen Atmosphere in a Medium Enriched with a VFA Mixture

**Figure 7:**
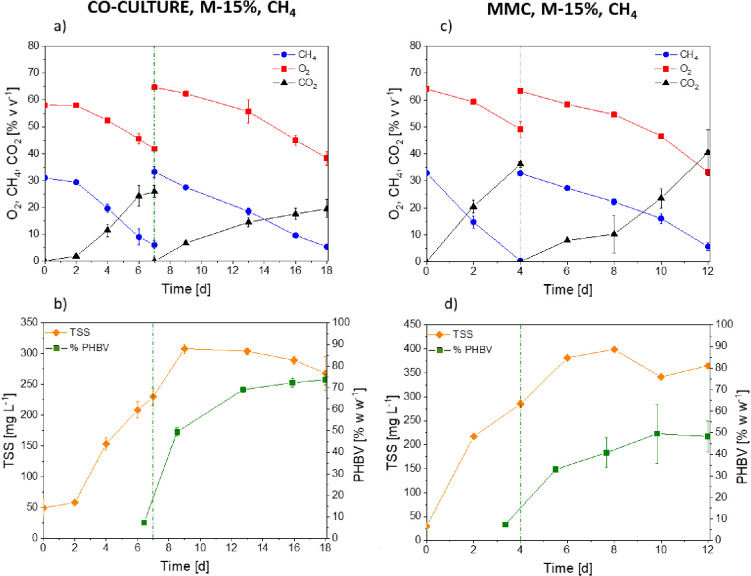
Time course of the oxygen, methane, carbon dioxide, TSS, and PHA concentrations in *M. parvus* + *R. opacus* cocultures (a,b) and mixed methanotrophic consortium (c,d) in a medium supplemented with VFA mixture (15% w w^–1^ of C_tot_).

The potential of VFA mixtures composed of valerate, propionate, acetate, and butyrate to replace pure valeric acid during the PHBV synthesis by *M. parvus* + *R. opacus* cocultures, and a mixed methanotrophic consortium was assessed during test series 2. The coculture (initial OD_600_ = 0.08) was grown up to 230 ± 14 mgTSS L^–1^ in the mineral salt medium by day 7, with concomitant use of 80.4 ± 5% v v^–1^ of CH_4_ and 28 ± 1.4% v v^–1^ of O_2_ initially present in the headspace (Figure 7a). The total net carbon dioxide production accounted for 383 ± 66 g m^–3^, which was the lowest obtained in this work with cocultures. On the other hand, the mixed microbial consortium (initial OD_600_ = 0.24) was grown up to a concentration of 285 ± 7 mgTSS L^–1^ by removing 99 ± 1.4% v v^–1^ and 23.2 ± 4.4% v v^–1^ of methane and oxygen initially fed by day 4. The total net carbon dioxide production was 285 ± 7 g m^–3^, which was the lowest observed for MMC in this work.

Cocultures of *M. parvus* and *R. opacus* were resuspended by day 7 in a nitrate-free medium, where a similar methane consumption of 84 ± 1.5% v v^–1^ was observed, while the uptake of oxygen increased up to 40.7 ± 4% v v^–1^ by day 18 (Figure 7a). The highest PHBV accumulation was detected by day 18 and accounted for 73.7 ± 2.5% w w^–1^ of PHBV (Figure 7b), with a PHB:PHV ratio of 94:6 (Figure 6). However, the largest PHV fraction was measured by day 1 and accounted for 20% of the total polymer produced. It should be highlighted that although the PHBV content was the highest observed in this work, the PHV fraction was slightly lower than that of the assays conducted with pure valeric acid and methane, likely due to a lower concentration of valeric acid supplied with the VFA mixture. Compared to the assays performed only with the VFA mixture, the simultaneous methane supply produced a PHBV content higher than those previously reported for *M. parvus*, thus confirming the enhancement obtained in cocultivation systems.

During the accumulation stage in MMC cultures, a maximum PHBV content of 49.6 ± 13% w w^–1^ was obtained by day 10 (Figure 7d), with a PHB:PHV ratio of 96:4 (Figure 6). The highest PHV content was detected by day 4 and accounted for 17.3%, thus suggesting that in the presence of VFA mixtures the production of PHV occurred during the first days of cultivation likely due to the preferential consumption of valeric acid. From day 5 onward, only PHB was synthesized. This trend in the generation of PHV, which was also observed in the cocultures, suggests that high copolymer contents during PHA production require only a few hours of starvation. Methane and oxygen consumption during the PHA accumulation phase in MMC accounted for 83 ± 4% v v^–1^ and 47 ± 4% v v^–1^, respectively, by day 12 (Figure 7c), while 796 ± 181 g m^–3^ carbon dioxide was produced (Figure 7c).

Finally, it is worth emphasizing that the PHBV yields achieved with MMC in this test series are higher than those previously reported in the literature for mixed microbial consortia, thus suggesting that the concomitant assimilation of methane and VFAs can enhance the performance of the culture.

### Test Series Comparison

The maximum biomass growth and PHB/PHV production rates obtained in all test series were compared and are reported in Table 2. Overall, the mixed consortium reached a higher growth rate than the synthetic cocultures in a time frame of 48 h. The use of valeric acid alone (no methane fed) resulted in a low PHV production rate for both cultures, while a low productivity of PHB was only reached using synthetic cocultures of *M. parvus* and *R. opacus*. The use of methane in cofeeding with valeric acid at 30% of the total carbon resulted, for both cultures, in the highest PHV production rate. Conversely, the use of the VFA mixture as 15% of the total carbon and methane as a gaseous substrate led to the highest PHB production rate for both synthetic cocultures and the mixed consortium. It can be highlighted that the use of a cosubstrate alone (no methane fed) is sufficient to produce PHBV only in the case of a very high purity cosubstrate (e.g., pure valeric acid), a condition which makes this process configuration costly and not suitable for industrial purposes. In this context, combining methane gas with a liquid cosubstrate increases the overall productivity and facilitates the replacement of pure valeric acid with renewable and cheaper cosubstrates, such as the mixtures of VFAs produced during the AD. Indeed, despite the production rate of PHV obtained with the VFA mixture being considerably lower if compared to the case of pure valeric acid used as a cosubstrate, there is a great potential for further investigation that requires attention. In this context, one of the strategies for optimizing the synthesis of 3-HV using renewable and biobased resources would be the enhancement of valeric acid production during the anaerobic digestion processes.^41^ Typically, digestate from AD mainly includes acetic, butyric, and propionic acids. However, prior reports have shown that adjusting the operating conditions during AD could also facilitate the production of valeric acid. For example, a reduction of the operating temperature from 55 to 45 °C and 35 °C was reported to increase the valeric acid fraction from 0% to 3.02% and 9.89%, while keeping the pH stable at 5.6 and 7 increased the valerate fraction from 0.15% to 9.5% and 3.6% respectively.^41^

**Table 2:** Maximum Growth Rate of Biomass and Maximum Production Rate of PHB and PHV Obtained for all Test Series

	synthetic coculture				methanotrophic enrichment			
maximum growth rate (biomass) and maximum production rate (PHB, PHV) [mg L^–1^ d^–1^]	VA-30%	VA-30% + CH_4_	M-15%	M-15% + CH_4_	VA-30%	VA-30% + CH_4_	M-15%	M-15% + CH_4_
biomass	36.3	33.8	25.8	47.7	61	92.3	76.1	93.6
PHB	4.3	40.3	0	62.6	0	30.5	0	50
PHV	7.5	35.7	0	5.1	10.2	21.8	0	2.4

## Conclusions

This study demonstrates the feasibility of VFA mixtures from mesophilic food waste fermentation for triggering poly(3-hydroxyvalerate) inclusion in synthetic cocultures and mixed methanotrophic consortia. The enhancements in PHA yields via cocultures are also demonstrated since the highest biopolymer content and PHV fraction were obtained by cultivating *M. parvus* and *R. opacus* under methane-enriched atmospheres. Finally, further studies devoted to optimizing the composition of VFA mixtures during PHBV production should be carried out to pave the way toward the scale-up of this green biotechnology.
